# Phosphoproteomic Analysis of Subcutaneous and Omental Adipose Tissue Reveals Increased Lipid Turnover in Dairy Cows Supplemented with Conjugated Linoleic Acid

**DOI:** 10.3390/ijms22063227

**Published:** 2021-03-22

**Authors:** Jayasimha Rayalu Daddam, Harald M. Hammon, Arnulf Tröscher, Laura Vogel, Martina Gnott, Gitit Kra, Yishai Levin, Helga Sauerwein, Maya Zachut

**Affiliations:** 1 Department of Ruminant Science, Institute of Animal Sciences, Agriculture Research Organization, Volcani Center, Rishon Lezion 7505101, Israel; Jayasimha@volcani.agri.gov.il (J.R.D.); gititk@volcani.agri.gov.il (G.K.); 2 Leibniz Institute for Farm Animal Biology (FBN), Institute of Nutritional Physiology “Oskar Kellner”, 18196 Dummerstorf, Germany; hammon@fbn-dummerstorf.de (H.M.H.); vogel@fbn-dummerstorf.de (L.V.); gnott@fbn-dummerstorf.de (M.G.); 3 BASF SE, 68623 Lampertheim, Germany; arnulf.troescher@basf.com; 4 Department of Animal Science, the Robert H. Smith Faculty of Agriculture, Food and Environment, the Hebrew University of Jerusalem, Rehovot 76100, Israel; 5 The Nancy and Stephen Grand Israel National Center for Personalized Medicine, Weizmann Institute of Science, Rehovot 7610001, Israel; Yishai.Levin@weizmann.ac.il; 6 Physiology Unit, Institute of Animal Science, University of Bonn, 53115 Bonn, Germany; Sauerwein@uni-bonn.de

**Keywords:** phosphoproteomics, adipose tissue, conjugated linoleic acid, endocannabinoid system, fatty acid synthase, lipid metabolism, dairy cow

## Abstract

Phosphoproteomics is a cutting-edge technique that can be utilized to explore adipose tissue (AT) metabolism by quantifying the repertoire of phospho-peptides (PP) in AT. Dairy cows were supplemented with conjugated linoleic acid (CLA, *n* = 5) or a control diet (CON, *n* = 5) from 63 d prepartum to 63 d postpartum; cows were slaughtered at 63 d postpartum and AT was collected. We performed a quantitative phosphoproteomics analysis of subcutaneous (SC) and omental (OM) AT using nanoUPLC-MS/MS and examined the effects of CLA supplementation on the change in the phosphoproteome. A total of 5919 PP were detected in AT, and the abundance of 854 (14.4%) were differential between CON and CLA AT (*p* ≤ 0.05 and fold change ± 1.5). The abundance of 470 PP (7.9%) differed between OM and SC AT, and the interaction treatment vs. AT depot was significant for 205 PP (3.5% of total PP). The integrated phosphoproteome demonstrated the up- and downregulation of PP from proteins related to lipolysis and lipogenesis, and phosphorylation events in multiple pathways, including the regulation of lipolysis in adipocytes, mTOR signaling, insulin signaling, AMPK signaling, and glycolysis. The differential regulation of phosphosite on a serine residue (S777) of fatty acid synthase (FASN) in AT of CLA-supplemented cows was related to lipogenesis and with more phosphorylation sites compared to acetyl-coenzyme A synthetase (ACSS2). Increased protein phosphorylation was seen in acetyl-CoA carboxylase 1 (ACACA;8 PP), FASN (9 PP), hormone sensitive lipase (LIPE;6 PP), perilipin (PLIN;3 PP), and diacylglycerol lipase alpha (DAGLA;1 PP) in CLA vs. CON AT. The relative gene expression in the SC and OM AT revealed an increase in *LIPE* and *FASN* in CLA compared to CON AT. In addition, the expression of *DAGLA*, which is a lipid metabolism enzyme related to the endocannabinoid system, was 1.6-fold higher in CLA vs. CON AT, and the expression of the cannabinoid receptor *CNR1* was reduced in CLA vs. CON AT. Immunoblots of SC and OM AT showed an increased abundance of FASN and a lower abundance of CB1 in CLA vs. CON. This study presents a complete map of the SC and the OM AT phosphoproteome in dairy cows following CLA supplementation and discloses many unknown phosphorylation sites, suggestive of increased lipid turnover in AT, for further functional investigation.

## 1. Introduction

Phosphoproteomics is a new, cutting-edge technique that can reveal the effect of a nutritional manipulation on the activity of proteins in a tissue [1]. To date, only a few studies have examined the phosphoproteome of adipose tissue (AT) in mammals; the global phosphoproteomic analysis of epididymal white AT revealed differential phosphorylation levels, as well as metabolic enzymes responsive to insulin in high-fat diets in mice [2,3]. In another study, the phosphoproteomics of white, beige, and brown AT has revealed a novel protein, casein kinase 2, as a negative regulator of beige adipocyte thermogenesis in rodents [3]. To the best of our knowledge, there is a lack of information about the difference in the proteomes of different white AT depots and about the AT phosphoproteome in cattle.

The functional properties of many biomolecules present in food and feed can alter lipid metabolism in AT. Conjugated linoleic acid (CLA) is a linoleic acid isomer (C18:2, *n*-6) that has been considered as an anti-obesity agent that is also useful in weight reduction. Research in rodents and humans has suggested that CLA could act to reduce adiposity through modulating properties in the lipid metabolism in AT [4,5]. However, the mechanism of CLA action in adipocytes is still unclear [6]. In dairy cows, CLA supplementation can be used to improve the energy deficit in early lactation by reducing the production of milk fat; such treatments were demonstrated to reduce the size of adipocytes in AT [7,8,9,10,11]. The influence of CLA was not found in the mobilization of body condition in early lactating cows [12]; however, it was suggested that CLA may inhibit lipogenesis in lactating cows [13]. In humans and rodents, different AT depots react differently to metabolic stimuli [14], and studies in dairy cows suggest that subcutaneous (SC) and internal AT depots may have different metabolic properties [15,16,17], therefore the response to CLA supplementation may be different in omental (OM) and SC AT. Generally, CLA reduces body fat by altering gene expression, inhibiting cell differentiation, and altering the activity of proteins involved in lipogenesis and lipolysis [18]. CLA in humans has shown minor effects in weight reduction and a high efficacy in metabolic diseases [19]. Studies have linked CLA with reduction in milk fat synthesis in dairy cows [20] and increased inflammation and insulin resistance in rodents and humans [21,22]. In adipocytes, CLA feeding regulates the gene expression of acyl CoA-synthetase, fatty acid synthase (FASN), and lipoprotein lipase (LPL) in lipid metabolism. The reduction in body fat is due to size reduction in adipocytes but not due to decrease in number of adipocytes [23]. Therefore, CLA supplementation would be expected to increase AT lipolysis, reduce the activity of LPL, and increase carnitine-palmitoyl-transferase-1 (CAT-1) activity in AT.

Functional differences in SC and OM fat depots were observed in rodents and humans, leading to various dissimilarities and the development of metabolic diseases [24,25]. Additionally, in dairy cows differences in functional alterations and changes in mobilization and accumulation was observed in SC and OM AT depots [26,27,28,29,30]. Therefore, we aimed to study the effects of CLA supplementation on the phosphoproteome of SC and OM AT in dairy cows. Our group was the first to define the proteome of AT in dairy cows [31], and has since analyzed AT proteomics in different conditions [32,33]; nevertheless, to date there has been no study on the phosphoproteome of AT in dairy cows. Hence, our objective was to explore the phosphoproteome of SC and OM AT in order to elucidate the mechanism of action of CLA supplementation on the phosphorylation of proteins in dairy cows. We conducted the label-free quantitative phosphoproteome profiling of AT in dairy cows to gain thorough molecular perception in AT following CLA supplementation. We identified site-specific phosphorylation sites on primary enzymes of insulin signaling and lipogenesis and lipolysis pathways, indicative of metabolic differences in lipid metabolism, via a detailed analysis of the modulated phosphoproteins.

## 2. Results

The phosphoproteomic strategy focused on quantitative mass spectrometry (MS) and was adopted to recognize and characterize changes in phosphorylation in dairy cows supplemented with conjugated linoleic acid (CLA) vs. control (CON), and also to compare the phosphoproteome of SC and OM AT.

### 2.1. Cows Performance, Blood Indicators and Carcass Adipose Tissue Measurements

The cows’ performance and blood metabolite parameters are shown in Table 1. The calculated energy balance of the CLA-supplemented cows was more positive than that of the controls (*p* = 0.04; Table 1), and the production of energy-corrected milk (ECM; *p* = 0.01), milk fat content (*p* < 0.001), and milk fat yield (*p* < 0.01) was much lower in CLA vs. CON cows; meanwhile, no differences in dry matter intake were observed among the groups (Table 1). When examining blood metabolites and hormones, the non-esterified fatty acids (NEFA) concentrations were lower in CLA vs. CON cows (*p* = 0.01; Table 1), while the concentrations of triglycerides, cholesterol, glucose, and beta-hydroxybuterate (BHBA) were similar among groups. The concentrations of insulin tended to be higher in CLA vs. CON cows (*p* = 0.07) and plasma glucagon tended to be lower (*p* = 0.06), while IGF-1 (*p* = 0.08) and IGFBP-3 (*p* = 0.06) tended to be higher in CLA vs. CON cows (Table 1). As expected, the plasma CLA content was significantly higher in CLA-supplemented cows when compared to the controls (Table 1).

Body weight (BW), hot carcass weight (HCW), cold carcass weight (CCW), adipose depot weights, and their proportion of BW at the slaughtering of cows are presented in Table 2. As shown, the BW, HCW, and CCW at slaughter were not different between groups, while the weight of OM adipose tended to be higher in CLA than in CON (*p* = 0.11), and the proportion of OM adipose as a percentage of BW was higher in CLA than in CON cows (*p* = 0.04; Table 2).

### 2.2. Phosphoproteomics Analysis of CLA-Supplemented SC and OM AT

**Figure 1:**
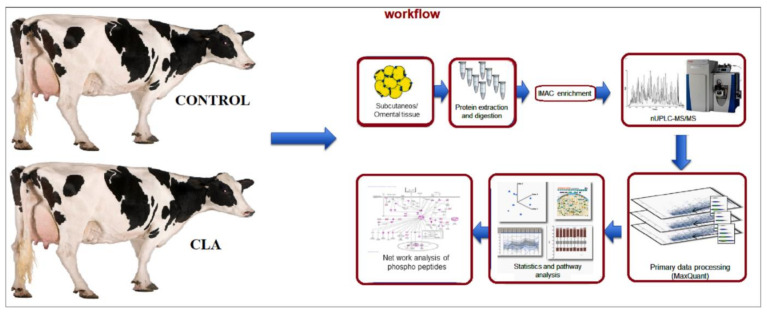
Workflow diagram of phosphoproteomic analysis by NanoUPLC-MS/MS of subcutaneous and omental AT from CLA-supplemented and control dairy cows. The subcutaneous (*n* = 10) and omental (*n* = 10) AT samples from 5 control and 5 CLA-supplemented cows were collected and protein was isolated. The extracted proteins were digested and the peptides were sequenced using nanoUPLC/MS/MS and phosphoprotome analysis. The primary data was quantified in Maxquant software and statistical values were corrected for pathway analysis. Network analysis was conducted using the cytoscape software related to lipid turnover. We detected a total of 5919 PP in AT at a 1 percent false localization rate (FLR). The abundance of 854 PP (14.4%) was different between CLA and CON (*p* ≤ 0.05, fold change (FC) ± 1.5) (Supplementary Data Sheet S1). The abundance of 470 PP (7.9%) differed between OM and SC AT (*p* ≤ 0.05, FC ± 1.5), and the interaction treatment vs. AT depot was significant for 205 PP (3.5% of total PP). From the 854 differential PP in CLA vs. CON, only 73 PP had a significant interaction of treatment vs. AT depot (8.5%; Supplementary Data Sheet S1). As the main effect observed was the difference between CLA and CON AT, we will first elaborate on these differential PP.

In this study, we identified phophopeptides using nanoUPLC-MS/MS from the SC and OM AT of CLA-supplemented cows and analyzed the ontology, pathways for lipid metabolism mechanism, and interactions involved in the network (Figure 1). The primary data obtained were processed using the MaxQuant software and the corrected *p*-values were used for the pathway analysis.

Increased protein phosphorylation—i.e., higher PP abundance—was found in several lipid metabolism proteins in CLA vs. CON AT (Supplementary Data Sheet S1): 8 PP were more abundant in acetyl-CoA carboxylase 1 (ACACA); 9 PP were more abundant in fatty acid synthase (FASN); 6 PP were more abundant in hormone responsive lipase (LIPE); 3 PP were more abundant in perilipin (PLIN); and 1 PP was more abundant in diacylglycerol lipase alpha (DAGLA), corresponding to 8438 unique PP. From these PP, the interaction treatment vs. AT depot was significant for 2 out of 9 differential PP in FASN (S2176; S2177 and S318) and for 1 out of 6 differential PP in LIPE (T567; Supplementary Data Sheet S1).

Approximately 80% (6748) of the differential PP in CLA vs. CON were single-phosphorylated peptides and only a small proportion, approximately 18.3% (1548) and 1.7% (142), were double- and triple-phosphorylated, respectively. The quantification of PP was performed using spectral count semi-quantitation, and we analyzed the differential expression with at least two-fold (10 percent FDR) upregulated phosphosites in response to CLA.

As mentioned above, the abundance of 470 PP (7.9%) differed between the OM and SC AT (*p* ≤ 0.05, FC ± 1.5). Increased protein phosphorylation was found in several lipid-metabolism proteins; 1 PP was higher in DAGLA, 1 PP in fatty acid binding protein 4 (FABP4), and 2 PP in FASN were higher in SC. vs. OM AT, while 1 PP in LIPE and 2 PP in perilipin-4 (PLIN4) were lower in SC compared to OM AT (Supplementary Data Sheet S1). The interaction between AT depot and treatment was significant for 1 PP in FASN (S318) and 1 PP in LIPE (T567) (Supplementary Data Sheet S1).

### 2.3. Analysis of Differentially Expressed Phophopeptides

**Figure 2:**
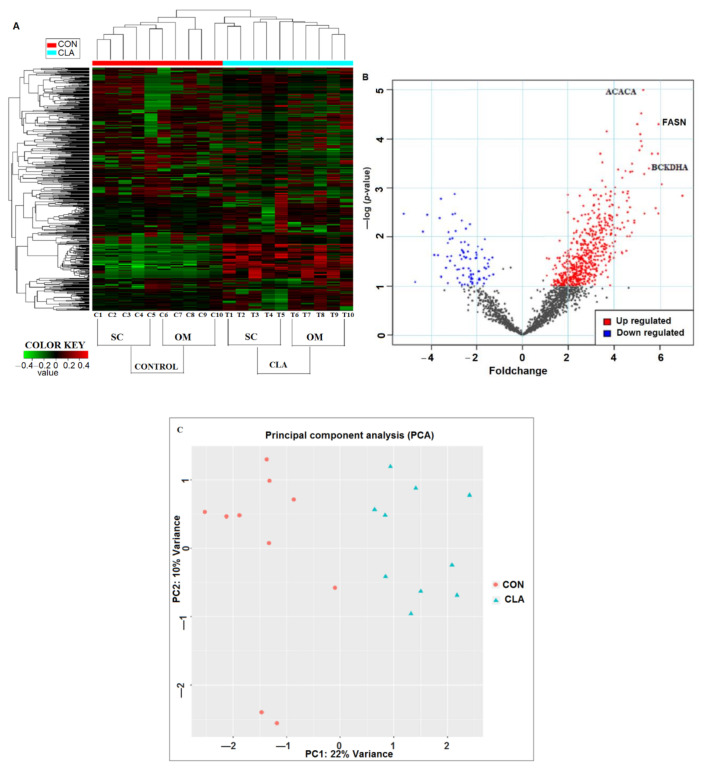
Differential expression analysis of phosphoproteome by IDEP 9.1 server using fold change and p-values of phosphopeptides. (**A**) Heat map analysis of significantly expressed phosphopeptides of control cow subcutaneous (SC) AT (C1–C5); control cow omental (OM) AT (C6–C10); CLA-supplemented cow SC AT (T1–T5); CLA supplemented OM AT (T6T10). The data represent the mass intensity of each peptide in the individual cows. Red boxes indicate upregulation, green boxes represent downregulation, and black boxes represent no change. (**B**) Volcano plot illustrates the significant expressed peptides of CON vs. CLA-supplemented dairy cows. The significant upregulated peptides are in red color and downregulated are in blue color. *X*-axis represents fold change and *y*-axis represented *p*-value. FASN-fatty acid synthase; ACACA-acetyl-CoA carboxylase 1; BCKDHA-branched-chain keto acid dehydrogenase E1 alpha. (**C**) Principal component analysis (PCA) of 22% variance in CON vs. CLA-supplemented SC and OM AT. PCA representing the separation of CON (red circles) with CLA-supplemented (blue triangles) samples.

The differential expression analysis of phosphoproteome data (using *p*-value (0.05) and fold change (±1.5)) in CLA vs. CON AT identified 854 differentially expressed proteins (Figure 2A–C). The K-means clustering of the differentially expressed proteins distinguished the SC and OM AT of CON- vs. CLA-supplemented dairy cows. The heat map showed the overall protein expression profile in individual CON- vs. CLA-supplemented dairy cows (Figure 2A). The CLA-induced phosphoproteome was divided into separate SC clusters (T1, T2, T3, T4, and T5) and OM clusters (T6, T7, T8, T9, and T10); both the SC and OM AT of CLA-treated cows showed an increased expression compared to the control (Figure 2A). In PP expression, the subsets of PP in control (C1 to C10) were minimally altered. In the clusters of the treated sample phosphoproteome, CLA induced an increase in phosphorylation when compared to control samples. The up- and downregulated peptides were identified by Volcano plots (pi-score, *p* < 0.05); Log-transformed *p* values (Student’s *t* test) associated with individual PP were produced against the log-transformed fold abundance shift between CON and CLA treatments. The upregulated peptides ACACA, FASN, and BCKDHA related to lipid turnover showed significant change in CLA-supplemented dairy cows (Figure 2B). Moreover, the principle component analysis of CON and CLA studied for the combination of OM and SC AT showed a variance of 22% change in the differential expression and was well separated, indicating the change in the expression of peptides in CON- vs. CLA-supplemented samples (Figure 2C).

### 2.4. Gene Ontology (GO) Analysis of Regulated Phosphoproteome

**Figure 3:**
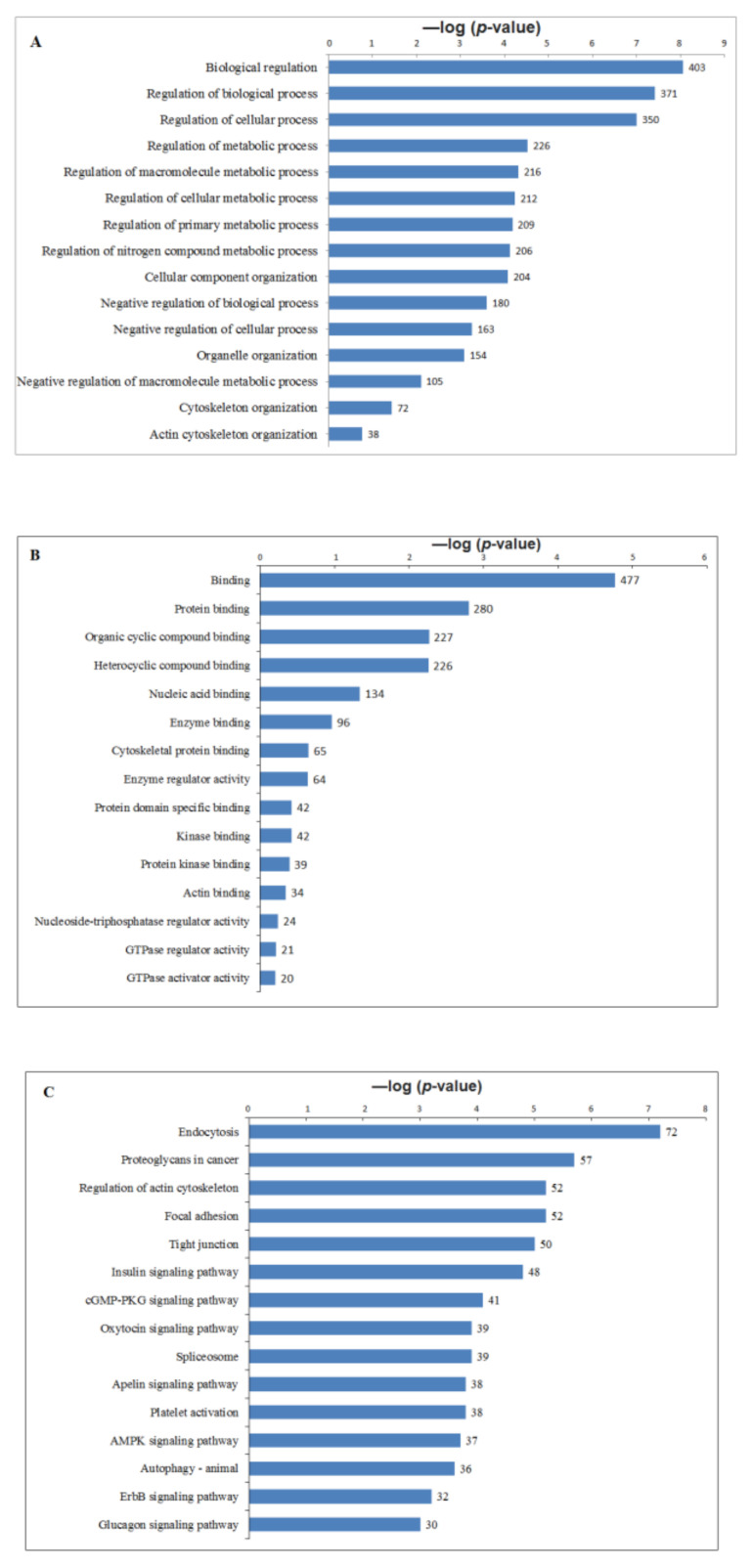
Significant altered phophopeptides were enriched by Gene ontology using BINGO in Cytoscape. Bar graphs summarizing the GO enrichment analysis, molecular function and pathways for the phopho peptides of CLA vs. CON subcutaneous and omental AT in dairy cows. The −log10 (*p*-value) on the *x*-axis and GO terms represented on *Y*-axis. (**A**) Top 15 significant GO terms associated with the phospho peptides in CLA vs. CON. (**B**) Top 15 molecular functions of the phospho peptides in CLA vs. CON. (**C**) Top 15 KEGG pathways enrichment analysis for the phospho peptides in CLA vs. CON. Number of peptides in each category was labelled on the bars.

GO enrichment analysis including biological process, molecular function and pathways of phophoproteins responsive to CLA supplementation was performed to understand the functional processes that were regulated in AT with increased phosphorylation levels (Figure 3A–C; Supplementary Data Sheet 2–4). The highlighted results showed different clusters enriched in CLA vs. CON, suggesting the unique signatures of AT phosphorylation on cows supplemented with CLA. Among the GO analysis, the regulation of biological process (Figure 3A), binding (Figure 3B) and endocytosis (Figure 3C) were most significant among phosphoproteome of SC and OM AT in dairy cows. In response to CLA, several proteins related to lipid turnover were found among top 15 in GO analysis, suggesting that these events of phosphorylation may be essential for metabolic regulation. Our findings support that phosphorylation-dependent signaling alters diet-induced metabolic pathways in CLA-supplemented cows. We studied and compared the biological processes, pathways, and molecular functions that were overrepresented in each cluster by gene ontology enrichment analysis to explore the biological functions associated with phosphoproteomes.

### 2.5. Kinase-Substrate Prediction

**Figure 4:**
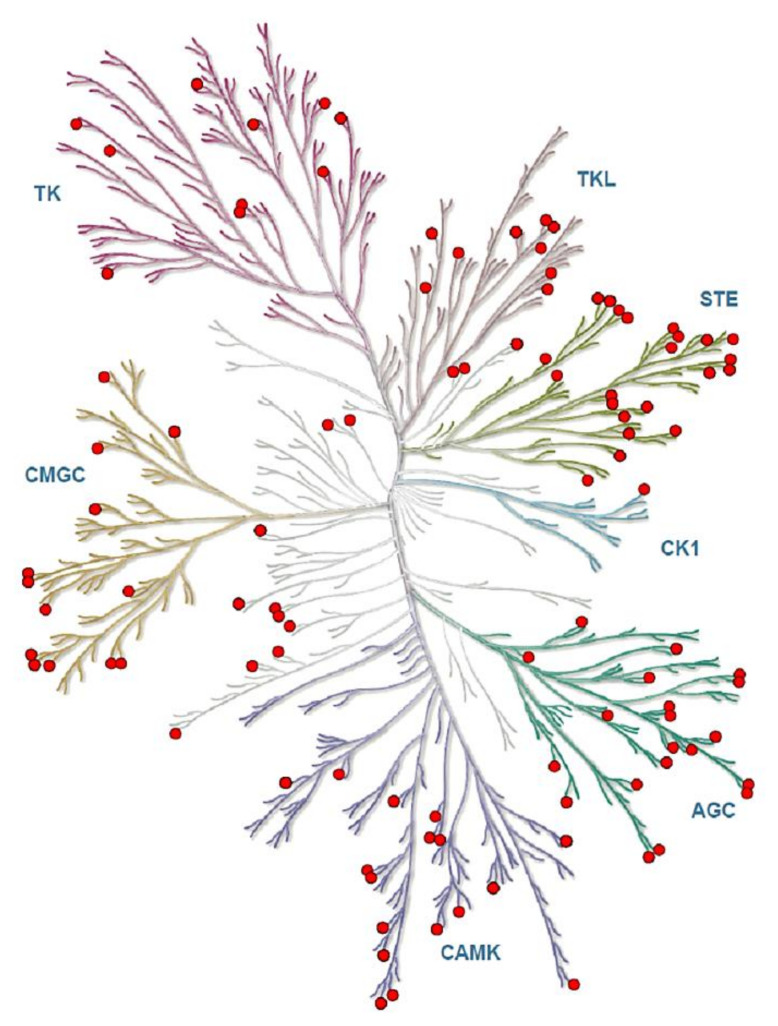
The predicted kinases of phosphoproteome in CLA-supplemented OM and SC AT were represented in kinome tree using the kinmap server. The identified kinases of phosphoproteome of SC and OM AT of dairy cows represents AGC: PKA/PKG/PKC-family kinases; CAMK: calcium/calmodulin-dependent kinases; CK1: casein kinases; CMGC: CDK/MAPK/GSK3/CLK-family kinases; STE: sterile homologue kinases; TK: tyrosine kinases; TKL: tyrosine kinase-like kinases. Phosphorylation sites of kinases are in Red circles. The kinases are separated as branches in the tree based on the category. Kinome tree Illustration reproduced courtesy of Cell Signaling Technology, Inc. (www.cellsignal.com, accessed on 12 December 2020).

The predicted up and down stream kinases for phosphorylated sites upon CLA supplementation in SC and OM AT give significant functional mechanisms, so we additionally mapped these protein kinases and obtained complete functional interactome (Figure 4). When we analyzed the distribution of phosphoserine, -threonine, and -tyrosine sites, we found Myotonin-protein kinase phosphorylation to be more frequent on protein kinases than on other identified proteins. Signaling response modulation depends on the complex protein networks and we used phosphoproteomic data to classify kinase activity in CLA-supplemented samples as a major molecular function, primarily aimed at identifying kinases that control uncharacterized metabolic pathway phosphorylation events (Supplementary Table S2).

### 2.6. The Analysis of Phosphoproteome Network

**Figure 5:**
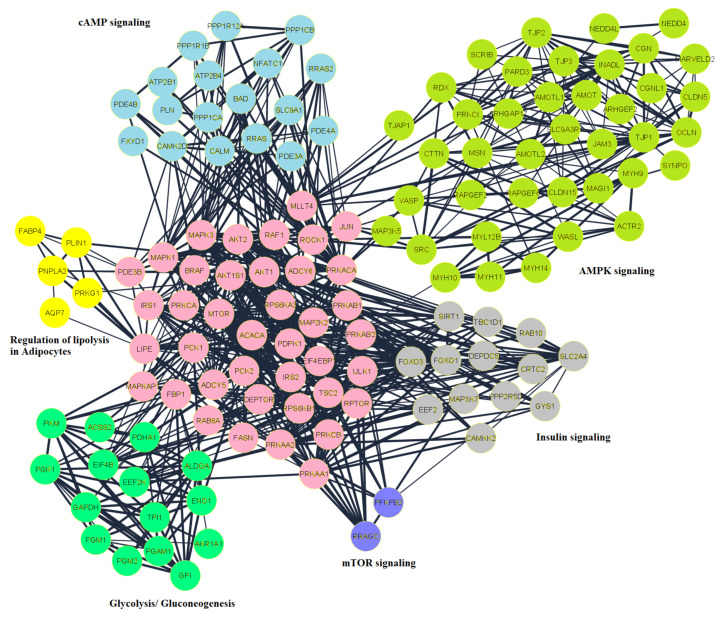
Network analysis of the peptides related to lipid turnover in SC and OM AT of CLA vs. CON was generated by Cytoscape software. The important networks of lipid turnover in adipocytes were developed using nodes of AMPK (light green), Insulin (grey), mTOR (purple), glycolysis (green). Acetyl-coA carboxylase-1 (ACACA), an important enzyme in the biosynthesis of fatty acids with seven upregulated phosphorylation sites, was noted in lipogenesis. Substantially upregulated was the main phosphorylation site at serine 78, indicating activation of ACACA in AT of cows supplemented with CLA. This phosphosite is a direct AMPK target and therefore matches well with the results of our kinase-substrate prediction that indicated reduced AMPK activity thus stimulating ACACA leading to lipogenesis in reaction to CLA supplementation. It is also important to note the observed downregulation of the phosphorylation of ABHD15, at serine 434 in our study, suggesting that this site may have regulatory mechanism in controlling the balance of lipids in lipid droplets.

An integrated functional connectivity of protein network was developed using STRING interactions, Reactome Functional Interaction, and Gene Go Metacore. Reactome FI and MetaCore interactions often reflect manually curated functional interactions informed by the pathway. In order to explore the molecular interactions of regulated phosphoproteins, both physical and functional association interactions were combined in this study. There were 243 nodes and 734 pairwise interactions in the interconnected network (Figure 5). Since the predicted upstream kinases also constitute important functional associations for each of the modulated sites on CLA, these AT interactions were also mapped to obtain a functional interactome. Examination of this detailed network’s topological parameters scale a free network which consider number of edges to one node. Some nodes had a greater number of edges than the whole network average and these ‘hubs’ are likely to represent key modulators. High connectivity was shown by approximately 33 nodes representing 15 percent degree of distribution, including kinases such as ADCY5 and ADCY6. AKT2, MAPK3, MTOR, PRKACA, and enzymes involved in fatty acid biosynthesis, ACACA, FASN and LIPE.

Network functional enrichment analysis revealed components of the pathway of fatty acid metabolism and enzymes are mapped onto the network. Using ClusterONE, linked protein clusters in the network were analyzed and five clusters out of six (*p* < 0.05) and one in six clusters with a *p* value of < 0.1 (Lipolysis in Adipocytes) were identified. Ontology/pathway research has evaluated the functions of each of these modules, and pathways related to lipid metabolism were clustered in cytoscape representing in different colors (Figure 5). The network analysis revealed the mechanism of altered PP during CLA supplementation, and suggested enrichment of lipolytic and lipogenic pathways. Among the clusters, the cluster shown in light green included AMPK signaling involved in the regulation of lipogenesis by controlling ACACA. This cluster included lipid metabolism components PRKACA, TJP1, and SRC, and found to be in close interaction with kinases, such as AKT1, AKT2, and MAPK3 (Supplementary Figure S1). The mTOR signaling components RPS6KA1, RPS6KA3, EIF4EBP1, and MTOR were found in the purple color cluster, and most of these acted as hubs in lipid metabolism. The components of the mTOR network contain highest number of hubs involved in lipid metabolism. Proteins involved in gluconeogenesis were depicted in the cluster shown in green. It is important to note that in AT of cows supplemented with CLA, most proteins in both these clusters exhibited phosphorylation. cAMP signaling network was in light blue color cluster with ROCK1, PLN, CALM, and SLC9A1. Regulation lipolysis in adipocytes components such as LIPE, FABP4, PNPLA2, AKT1, and ADCY5 were present in the cluster shown in a yellow color. Insulin signaling pathway components such as FOXO, PCK, and AKT were found to also control ACACA in the cluster of grey color in close proximity to proteins (Supplementary Figure S2). The gluconeogenesis pathway includes important components of lipid metabolism such as ACSS2, PDHA1, and PKM shown in a green color. Therefore, our network review highlighted the connection of components that were important in collaborating to construct cellular physiology and metabolic process.

**Figure 6:**
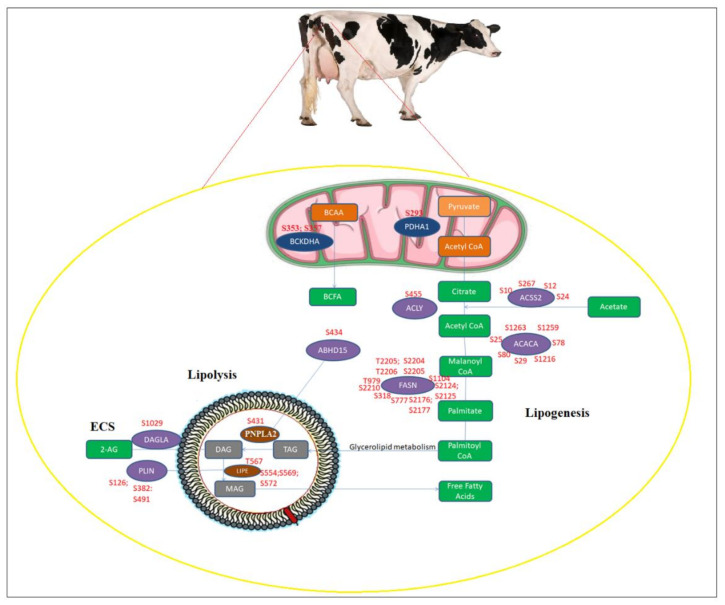
Altered phosphorylation of lipid metabolic pathways including lipogenesis and lipolysis in SC and OM AT of dairy cows supplemented with CLA. Enzymes with increased or decreased phosphorylation upon CLA supplementation were mapped to lipid metabolism networks. Additionally, the endocannabinoid system (ECS) mechanism was linked to lipolysis based on the literature. Enzymes and metabolites were represented in circles and rectangles respectively. Altered enzymes in cytoplasm shown (purple), in mitochondria (dark blue); and in lipid droplets (brown); metabolites of lipogenesis in mitochondria (orange) and in cytoplasm (green); metabolites of lipolysis in lipid droplets (grey color).

In Pyruvate Dehydrogenase E1 Subunit Alpha 1 (PDHA1), upregulated phosphosite at S293 was observed and the phosphorylation of this site increases the activity of the enzyme, thus increasing the production of mitochondrial acetyl-coA; this can be directed to cytoplasm to store it as fat or undergo oxidation in mitochondria (Figure 6). Several sites with phosphorylation on acetyl-coenzyme A synthetase (ACSS2) in cytoplasm have also been observed, mediating the development of cytoplasmic acetyl-coA which is important in the synthesis of fatty acids. We observed that ACLY S455 led to the phosphorylation of AKT, indicating that this phosphosite plays an important role in controlling the signaling of insulin. Several changes to acetyl-coA-forming enzymes in cytoplasm lead to increased ACACA activity, indicating the possibility of increased biosynthesis of fatty acids in adipocytes.

In addition, fatty acid synthase (FASN) phosphosite abundance, which is the second fatty acid biosynthesis enzyme in sequence following ACACA, was substantially altered upstream of ACACA. Threonine 2206, this particular phosphosite, emphasizes its major role in the specific functions of adipocytes. In addition to these lipogenic enzymes, branched-chain keto acid dehydrogenase E1 alpha (BCKDHA) phosphorylation was observed, which catabolizes branched-chain amino acid (BCAA) and also in branched-chain fatty acid (BCFA) synthesis, controlling BCAA metabolism.

With increased phosphorylation, the main proteins participating in the lipolysis were also observed in AT (Figure 6). In addition, the reduced phosphorylation of hormone sensitive lipase (LIPE) was observed at a novel site. The enzyme has potential implications for increasing, trapping and routing the absorption of fatty acids for downstream lipid metabolism. Indeed, at the key regulatory site operation, threonine 567 and serine 554, 569, 572, we observed phosphorylation of rate-limiting LIPE, suggesting an increase in lipolytic activity of LIPE in AT during CLA supplementation. A significant phosphorylation of adipose triglyceride lipase (PNPLA2) was also observed at Serine 431. Considering these changes in kinases in signaling mechanisms during CLA supplementation, our study demonstrates a change in the lipogenesis and lipolysis pathways balance in AT.

### 2.7. Gene Expression in CLA vs. CON AT

When analyzing SC and OM AT together, the mRNA abundance of the lipid metabolism enzymes *LIPE* (*p* = 0.03) and *FASN* (*p* = 0.01) was higher in CLA compared to CON AT, while no differences in the expression of *PLIN* were observed (Table 3). The relative expression of the lipid metabolism enzyme *DAGLA*, that is also related to the endocannabinoid system (ECS), was 1.6-fold higher in CLA vs. CON AT (*p* = 0.05), while the expressions of the other ECS enzymes *FAAH, NAPEPLD* and *MGLL* were not different between treatments (Table 3). The mRNA expressions of the cannabinoid receptor CB1 (*CNR1*) were reduced by 39% (*p* = 0.04), and the expression of the cannabinoid receptor CB2 (*CNR2*) tended to be lower (*p* = 0.08) in CLA vs. CON AT (Table 3). The effect of the AT depot was significant for *PTGS2* (*p* = 0.04), *DAGLA* (*p* = 0.05), *MGLL* (*p* < 0.0001), *FAAH* (*p* = 0.002), *CNR1* (*p* = 0.02) and *CNR2* (*p* < 0.0001; Table 3); the relative expressions of *CNR1, CNR2, MGLL, DAGLA* and *PTGS2* were higher in SC than in OM AT, while the expression of *FAAH* was higher in OM than in SC AT. The interaction of treatment (CLA vs. CON) and AT depot was not significant for any of the examined genes (Table 3). The gene expression in each AT depot is shown in Supplementary Table S3; in OM, the average relative expressions of *LIPE* (*p* = 0.02) and *DAGLA* (*p* = 0.03) were higher in CLA than in CON, and the expression of *FASN* tended to be higher in CLA than in CON (*p* = 0.06). In SC AT, the expression of *FASN* tended to be higher in CLA than in CON (*p* = 0.06), while the expressions of *PTGS2* (*p* = 0.07), *CNR1* (*p* = 0.06) and *CNR2* (*p* = 0.08) tended to be lower in CLA than in CON (Supplemental Table S1).

### 2.8. Immunoblots of CLA vs. CON AT

**Figure 7:**
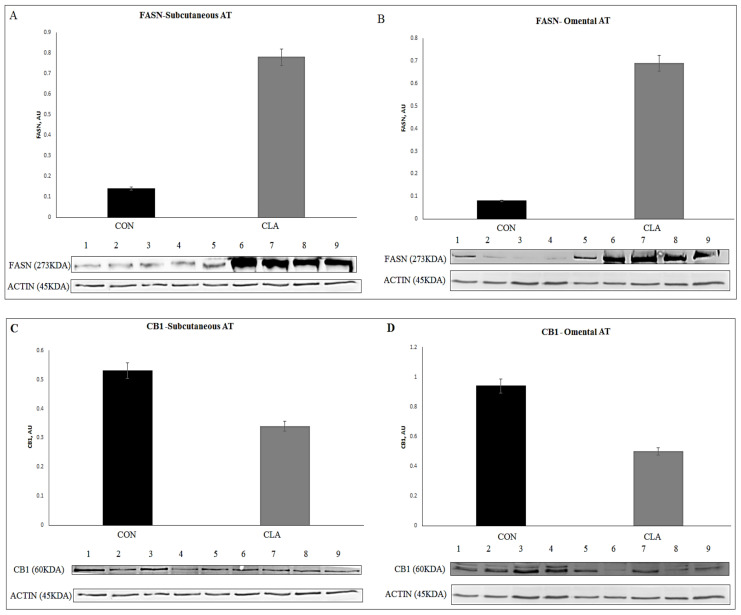
Immunoblot analysis of lipogenesis was measured by the abundance of FASN in CON vs. CLA (*n* = 1–4 replicates for CON; *n* = 5–9 replicates for CLA). (**A**) Abundance levels of FASN in subcutaneous (SC); (**B**) abundance levels of FASN in omental (OM) AT in CLA and CON. Immunoblot analysis of the endocannabinoid receptor CB1 abundance in CON vs. CLA (*n* = 1–4 replicates for CON; *n* = 5–9 replicates for CLA). (**C**) Abundance of CB1 in subcutaneous (SC); (**D**) omental (OM) AT CLA and CON. Actin was used as a control to quantify the proteins.

The total abundance of several proteins related to lipid metabolism in AT of CLA and CON cows were examined in SC and OM AT (Supplementary Table S4 and Figure S4A,B); in SC AT, the average abundance of FASN was higher in CLA than in CON (*p* = 0.01; Figure 7A), while CB1 was lower (*p* = 0.02; Figure 7C) in CLA than in CON. In OM AT, the average abundance of FASN was higher (*p* = 0.01; Figure 7B) and the abundance of DAGLA tended to be higher (*p* = 0.09) in CLA than in CON, while the average abundances of CB1 (*p* = 0.006; Figure 7D) and FAAH (*p* = 0.009) were lower in CLA than in CON. When analyzing SC and OM together, an increased total abundance of FASN (6.6-folds; *p* = 0.0004) was found in CLA vs. CON AT. In addition, the abundance of CB1 was 42% lower in CLA AT (*p* = 0.0003).

## 3. Discussion

In the present study, we have established the first database of phosphoproteome from the SC and OM AT of dairy cows, containing phosphorylations of ~5900 PP. This dataset can be used as a reference for further studies on the AT phosphoproteome and add important new insights into AT physiology in dairy cows. Phosphoproteomics analysis demonstrated the effects of the nutritional supplementation of CLA during early lactation on the phosphoproteome of OM and SC AT. Overall, we found an enrichment of the lipid metabolism pathways in AT, specifically lipogenesis and lipolysis, which could suggest an increased lipid turnover in AT and explain the metabolic response to CLA supplementation; these data add important new information on specific PP that are affected by CLA.

In dairy cows, research on CLA supplementation has focused primarily on milk fat depression, increased milk yield, energy metabolism, and antioxidative and inflammatory response [8,9,10,11,20]. Nevertheless, the effects of dietary CLA (mainly using cis9-trans11 and/or trans10-cis12 CLA isomers) indicate systemic metabolic effects. Rumen-protected CLA decreased milk fat, increased milk production, and improved the tissue energy balance of dairy cows during in the first 15 weeks of lactation [20,34]. The size of adipocytes decreased in the long-term study in dairy cows after CLA supplementation, indicating an effect on different fat depots [7], a reduction in endogenous glucose production and milk fat during early lactation [35], a reduction in circulating adiponectin concentration in primiparous and multiparous cows [36], a decreased milk fat content and yield, a slightly increased milk yield, and a marginal decrease in DMI [8]. Recently, we demonstrated that CLA supplementation elevates the plasma glucose and insulin concentrations and stimulates the somatotropic axis in cows [37]. In a comprehensive companion study, we found that cows supplemented with CLA have a lower milk fat production, an improved energy balance, less BW reduction postpartum, and had more body and omental fat than control cows at slaughter (63 days in lactation) [31]. In this study, we collected and examined the SC and OM AT from a subset of 10 cows that were randomly selected from a comprehensive study [38] and similarly found a lower ECM and milk fat production and an improved energy balance in CLA vs. CON cows. Moreover, we found lower plasma NEFA concentrations and a higher proportion of omental fat as a percentage of BW in CLA vs. CON, which is in accordance with the findings of Vogel et al. [38]. In the AT, there is a constant balance between anabolic (lipogenesis) and catabolic (lipolysis) processes in order to maintain energy homeostasis, as AT serves as an important source of energy in the postpartum dairy cow. Based on these effects of CLA, it seems that the CLA-supplemented cows had an increased rate of lipogenesis relative to lipolysis in AT, at least in the OM adipose depot. We suggest that the significant decrease in energy output with milk, due to the inhibitory effect of CLA on milk fat production, has enabled the CLA-supplemented cows to store more energy in their AT in early lactation compared to the CON.

In order to explore the molecular mechanism of the effects of CLA supplementation on AT in dairy cows, we performed a phosphoproteomic analysis of OM and SC AT from the control and CLA-treated groups. In our study, the main effect on the phosphoproteome was of the CLA supplementation, therefore we considered SC and OM AT together for the AT prediction of kinase-substrate and the analysis of the integrated network of altered phosphoproteins, showing the underlying mechanisms deregulating the lipogenesis and lipolysis pathways. We investigated the effect of cis9-trans11 and trans10-cis12 CLA isomer supplementation on the phosphoproteome of AT depots in dairy cows, and found a total of 5919 PP in AT, with 854 PP being between CLA and CON. Increased protein phosphorylation was found in lipid metabolism proteins (ACACA, FASN, LIPE, PLIN) in CLA vs. CON AT, indicating the enrichment of lipid metabolism in the AT of CLA cows. In male leptin-deficient (ob/ob) mice, the nutritional supplementation of cis9-trans11 CLA affected the white AT proteome by inducing the differential regulation of 43 proteins that are related to redox status and modulated mechanistic links between the actin cytoskeleton, insulin signaling, glucose transport, and inflammation in AT [39]. Another study conducted proteomic analyses and revealed that trans10-cis12 CLA showed pro-atherogenic effects and induced insulin resistance in atherosclerosis-prone apolipoprotein E-deficient (Apoe^−/−^) mice, while cis9-trans11 CLA was anti-atherogenic and induced heat shock protein 70 kDa expression [40]. Very few data are available in dairy cows that indicate changes in insulin response in AT due to CLA supplementation. In mice, the daily supplementation of trans10-cis12 CLA reduces white AT and adiponectin, leptin, and serum levels, as well as initiating hyperinsulinemia [41]. Moreover, it directly induces inflammatory gene expression in adipocytes as well as macrophage infiltration into AT into a local inflammative state, contributing to insulin resistance [42]. Enhanced CLA diets lead to rapid and significant fat depletions in several species, including pigs, rats, hamsters, chickens, and mice [43,44,45]. Trans10-cis12 CLA may cause an effect by increasing energy consumption, fecal loss, apoptosis, the oxidation of fatty acids, and lipolysis and decreasing preadipocyte differentiation and lipogenesis [43]. In this regard, the effects of CLA that were observed in our study and in Vogel et al. [38] of increased AT and a possibly higher rate of lipogenesis rather than lipolysis in the AT of dairy cows are quite surprising. We propose that in dairy cows, CLA promotes lipolysis to some extent, and in addition the reduction in the energy output in milk allows for increased lipogenesis, therefore the overall effect of CLA supplementation on AT metabolism is by increasing both lipolysis and lipogenesis simultaneously. It should be noted that the CLA dosages used in rodent and human studies are much higher than those supplemented in the present experiment, which could also contribute to the unique response on the AT of the dairy cows in this study. Overall, it seems that CLA has a unique effect on the AT of dairy cows, and the specific effect of reduction in milk fat is responsible for this lipogenic effect in the AT of dairy cows.

In the present study, the changes in the phosphoproteome caused by CLA were studied by the k-means clustering of the differentially expressed PP. Among the differentially abundant PP, ACACA, MAP4, ENAH, FASN, SLC16A7, AKAP12, DDHD2, and BCKDHA were significant in AT, that were related to insulin signaling, lipolysis in adipocytes, MAPK signaling, gluconeogenesis, and the mTOR signaling pathways. Lipid metabolism includes the following processes: lipolysis, fatty acid oxidation, lipogenesis, cholesterol synthesis, and ketone body synthesis (Supplementary Figure S3). Lipogenesis is manifested by the aberrant trafficking of acetyl CoA in mitochondria and dependent on PDHA1 phosphorylation. In cytoplasm, acetyl CoA is formed from the precursors acetate and citrate by ACSS2 and ACYL, respectively. We identified ACSS2 and ACYL with elevated phosphorylation in CLA AT, suggestive of an upregulation in acetyl CoA formation and subsequent import in the AT of cows with CLA supplementation. In addition, we observed the enrichment of AMPK signaling, which is known to be a key modulator of lipogenesis. Insulin signaling, cAMP signaling, and mTOR signaling components were high in number and also showed altered phosphorylation sites with CLA treatment (Supplementary Table S2). In addition, we have also observed the phosphorylation of ACACA, a recognized lipogenesis regulator. From the bioinformatics analysis of AT, it was observed that AMPK signaling is the one of the top pathways of the phosphoproteome, which controls the mechanism of ACACA and the rate of lipogenesis. With regard to the phosphorylation events of lipolysis, PNPLA2 was upregulated with phosphorylation at the site serine 431, and this is the initiating step of lipolysis, where TAG is converted to DAG. Interestingly we observed the downregulation of LIPE and hence the conversion of DAG to MAG is reduced, which leads to the more abundant availability of DAG in lipid droplets of adipocytes. We also observed the upregulation of DAGLA with phosphosite at serine 1029, which is related to the ECS by converting DAG to the main endocannabinoid 2-AG.

The phosphoproteome representing kinase signaling events exposed activated or suppressed enzymes in lipid metabolism by connecting altered events in the sense of metabolic networks. In this work, upstream kinases were predicted that are possibly involved in the lipid metabolism of AT in dairy cows during CLA supplementation using our dataset. With the studies that have observed based on the diet-induced insulin regulation, our prediction resonates well. In our study, CLA-supplemented cows had increased the phosphorylation of kinases in AT, which led to other pathways such as adhesion, tight junction, and endocytosis apart from lipid metabolism. Lipolysis occurs by beta-adrenergic receptors by catecholamine and the subsequent activation of protein kinase A (PKA) [46]. LIPE and perilipin A are the two main lipolytic targets of PKA in adipocytes. Upon PKA activation, phosphorylated LIPE translocates to the lipid droplet where LIPE hydrolyzes triglycerides. In addition, the activation of lipolysis is also strictly dependent on the PKA-mediated phosphorylation of perilipin A. Lipogenesis and lipolysis are controlled to preserve the energy balance in order to respond to different nutritional states [47,48]. We observed phosphorylation in our dataset on lipogenesis enzymes, including ACACA, ACSS2, and FASN with CLA supplementation. Cytoplasmic acetyl-CoA accumulation primes fatty acid synthesis, and also we observed phosphorylation on ACLY and ACSS2, important enzymes in lipogenesis. We assume that the concerted effect of such phosphorylation on metabolic enzymes can lead to metabolic change and an increase in the levels of triglycerides. Indeed, at serine 10 we found a novel phosphosite on ACSS2, essential for regulating the balance of lipogenesis and lipolysis. We recognized lipolysis and lipogenesis components from our network, establishing close links with each other, as well as with main metabolic kinases such as AKT1, PKA, and AMPK, indicating possible regulation among the different pathways. Thus, the phosphorylation on lipogenesis enzymes, including ACSS2, along with the altered kinase signaling peptides leads us to confirm the increase in lipogenesis after CLA supplementation in AT. In addition, in the present study we found an increased mRNA expression of LIPE and FASN and an increased protein abundance of FASN in CLA vs. CON AT. CLA supplementation has direct links with milk fat depression by regulating milk fat synthesis regulators in cattle; it was found that the supplementation of CLA reduced milk fat by reducing the PPARγ2 regulator in the mammary glands [49]. The increase in FASN supports the lipogenesis process that we describe based on the bioinformatics as well as the improved energy balance, lower blood NEFA, and tendency for a higher proportion of OM AT in CLA-supplemented cows. Though the abundance of proteins related to lipid metabolism in the AT of CLA and CON cows was examined, we found an increased total abundance of FASN (6.6-fold; *p* = 0.0004) in CLA vs. CON AT. Overall, the effects of CLA were mostly similar in SC and A AT, as minor interactions between AT depot and dietary treatment were observed in the differential PP from the lipid metabolism-related proteins in this study. The mechanisms of the phosphoproteome can be understood by analyzing the phosphosites and phosphopeptides at an in-depth level [50]. This research enhances the importance of CLA supplementation in the lipid metabolism of AT by offering a study of the changed phosphorylation profile at the molecular level. The CLA supplementation increased the lipid turnover by stimulating lipolysis in SC and OM AT of dairy cows and on the other hand stimulating lipogenesis to store energy that is not used for milk fat synthesis. Our global phosphoproteome profile emphasizes metabolic regulation based on phosphorylation and also reveals many uncharacterized phosphorylation sites that play important roles in the metabolic pathways of lipids.

Due to the great importance of the ECS in AT metabolism [51], as well as our finding of the upregulation of DAGLA with phosphosite at serine 1029, we were interested in examining the effects of CLA supplementation on the ECS in AT. In the present study, the relative gene expression of DAGLA, which is a lipid metabolism enzyme also related to the ECS, was 1.6-fold higher in CLA vs. CON AT. The ECS is a central regulator of mammalian metabolism and energy homeostasis; it controls the energy balance by controlling appetite [52,53], intracellular lipolysis, and energy expenditure. Recently, we demonstrated that the ECS is involved in AT metabolism in postpartum dairy cows [18]. To this end, we found that both the mRNA expression and protein abundance of the cannabinoid receptor CB1 were reduced in CLA vs. CON AT. The ECS contains endocannabinoids and receptors, ligands, and enzymes synthesizing endocannabinoids. CB1R (CB1) and CB2R (CB2) are G-coupled protein cannabinoid receptors. CB1 is expressed centrally and in white AT, and CB2 is expressed predominantly in immune cells [54]. In non-ruminants, upon ligand binding to CB1 lipogenesis is enhanced by three mechanisms: the promotion of LPL activity, the inhibition of AMPK, and the augmentation of insulin-dependent glucose uptake. We have previously demonstrated that the mRNA expression of CB1 in AT tended to be greater in cows exhibiting high rates of lipolysis, suggesting that CB1 may be related to an increased NEFA content in AT [18]. In adipocytes, the CB1 receptor stimulation increases the uptake of glucose and lipogenesis while inhibiting lipolysis. Dietary CLA has been shown to decrease the levels of the endocannabinoid 2-arachidonoyl-glycerol (2-AG) in the cerebral cortex of mice [55], and rats fed with 1% CLA (mixture of c9,t11 and t10,c12 isomers) had increased levels of the endocannabinoids N-oleoylethanolamide (OEA) and N-palmitoylethanolamide (PEA) in the liver. In dairy cows, dietary CLA decreased the mRNA expression of the CB2 in the uterus [56]. Based on this, we suggest that the lower expression and abundance of CB1 in the AT of the CLA-supplemented cows may be a negative feedback to the lipogenic signals that were induced following CLA supplementation in order to control AT lipogenesis; however, this issue requires further investigation. Interestingly, we found that the relative gene expressions of PTGS2, DAGLA, MGLL, CB1, and CB2 were higher in SC than in OM AT, while the expression of FAAH was lower in SC than in OM AT. This may indicate that the ECS tone is differential between these AT depots, however further studies should be conducted to elucidate this issue.

## 4. Materials and Methods

### 4.1. Animals and Design

All the experimental procedures were carried out in accordance with the German Animal Welfare Act and were approved by the relevant Department for Animal Welfare Affairs of the state of Mecklenburg-West Pomerania (Landesamt fur Landwirtschaft, Lebensmittelsicherheit und Fischerei Mecklenburg-Vorpommern, Germany; LALLF M-V/TSD/7221.3–1-038/15). The cows were kept in a free stall barn at the Leibniz Institute for Farm Animal Biology (FBN), Dummerstorf, Germany. This work was part of a comprehensive study described in Vogel et al. [38]. In short, rumen cannulated Holstein cows (*n* = 10) that were randomly selected from the experimental groups were supplemented with corn silage-based TMR and daily abomasally supplemented with coconut oil (CON, *n* = 5; 45.5% C12:0; 16.9% C14:0; 76 g/d) or Lutalin^®^ (CLA, *n* = 5; c9, t11 and t10, c12, 10 g/d) from wk 9 prepartum and after slaughtering at 63 d postpartum (Supplementary Tables S5 and S6). At slaughter, omental (OM) and subcutaneous (SC) AT were collected as described in Vogel et al. [38] and frozen (−80 °C).

### 4.2. Blood Samples for Indicators of Metabolic State

Blood samples were taken on d 63, 42, 35, 28, 21, and 10 before expected calving; on d 1; and then once weekly up to d 56 after calving immediately after morning milking before supplementation, via jugular vein puncture using the Vacuette system (Greiner Bio-One International GmbH, Kremsmunster, Austria). Samples were immediately placed on ice and centrifuged within 30 min at 1565× *g* at 4 °C for 20 min and the collected plasma was stored at −20 °C until further analysis. Evacuated tubes containing sodium fluoride in combination with potassium oxalate as an anticoagulant were used to measure the plasma concentrations of glucose, triglycerides (TG), nonesterified fatty acids (NEFA), β-hydroxybutrate (BHB), and total cholesterol (TC). Plasma metabolites were analyzed using an automatic spectrophotometer (ABX Pentra 400; Horiba) and respective kits: NEFA, #434 91,795 (acyl-CoA synthetase—acyl-CoAoidase method) from Wako Chemicals GmbH (Neuss, Germany); TG, #A11A01640 (lipoprotein lipase–glycerin kinase−glycerin-phosphate-oxidase method), and TC, #553-126 (cholesterol oxidase method) from mti Diagnostics GmbH (Idstein, Germany); glucose (#A11A01667, hexokinase–glucose-6-phosphate dehydrogenase method; HORIBA ABX SAS, Montpellier, France) and BHB (#RB 1008, 3-hydroxybutyrate dehydrogenase method; Randox Laboratories Ltd., Crumlin, UK). The cis-9, trans-11, and trans-10, cis-12 CLA in plasma was measured in blood samples containing EDTA (K3EDTA, 1.8 g/L) at d −63, −42, 1, 28, and 56 relative to calving [57].

Concentrations of hormones in plasma were analyzed in blood samples from evacuated tubes containing EDTA (K3EDTA, 1.8 g/L). The concentrations of plasma insulin (#RIA-1257) and glucagon (#RIA-1258) were determined via RIA using kits from DRG Instruments GmbH (Marburg, Germany) adapted for cattle [58]. The intra- and interassay coefficients of variation were 3.7 and 5.5% for insulin and 4.6 and 13.4% for glucagon. Plasma cortisol concentrations were analyzed using a commercially available ELISA kit (#EIA1887; DRG Instruments GmbH, Marburg, Germany) according to the manufacturer’s instructions. The assay was validated for use with bovine plasma [59]. The test sensitivity was 3.5 ng/mL, and the intra- and interassay coefficients of variation were 4.7 and 12.7%, respectively. Plasma growth hormone (GH) and insulin-like growth factor (IGF)-I were measured by radioimmunoassay, as described previously by Vicari et al. and Vogel et al. [37,60]. Intra- and interassay coefficients of variation for GH and IGF-I RIA were below 10 and 15%, respectively. The concentrations of plasma IGFBP were analyzed via quantitative Western ligand blot analysis as previously described using plasma samples containing K3-EDTA [37,61]. The intra-and interassay coefficients of variation were <15% and <20.0% for all IGFBP, respectively [37]. The plasma concentrations of leptin and adiponectin were analyzed in EDTA-plasma samples by using an ELISA established by Sauerwein et al. [62] and Mielenz et al. [63] and modified by Kesser et al. [42], respectively. The inter- and intraassay variation coefficients were 7.8% and 3% for leptin and 13.7% and 4.9% for adiponectin.

### 4.3. Sample Preparation for Phosproteome Analysis

All the chemicals used in this study are from Sigma Aldrich (Rehovot, Israel), unless stated otherwise. Samples were lysed with 8 M urea 0.1 M Tris/HCl pH 8.0 buffer supplemented with phosphatase inhibitors (Sigma, P0044), and then subjected to in-solution tryptic digestion. Lysate centrifuged for 10 min at 16,000× *g* and clear lysate was transferred to a fresh tube. Proteins were reduced by 5 mM DTT for 50 min at 50 °C and alkylated with 10 mM of iodoacetamide for 20 min at room temp in the dark. The samples were diluted 1:4 using 50 mM Ammonium bicarbonate and digested with trypsin (1:50 ratio) at 37 °C for overnight and a second trypsin digestion for 4 h at 37 °C. Digestion was ended by adding trifloroacetic acid to 1% concentration, desalted by Oasis HLB in μElution format (Waters, Milford, MA, USA) and then vacuum dried and stored in −80 °C until further analysis.

For phosphoproteomics, phosphopeptides were enriched using a ProPac IMAC-10 column (4 × 50 mm) (Thermo Fischer Scientific, San Jose, CA, USA) from the total protein digest. Peptides were loaded with the flow 0.1 mL/min on the column in 10 min (0% B). Phosphopeptides have been eluted from the column with the following gradients: first 10 to 15 min elution with flow of 0.6 mL/min (B between 0 and 15%), second 15–44 min elution with a flow of 0.02 mL/min (B between 15 and 30%); wash with flow of 1 mL/min (B from 30 to 50%) from 44 min to 50 min; then balance from 50 to 60 min with flow of 1 (0% B). The second elution of the phosphopeptides were collected in 1.5 mL (~1.2 mL), vacuum to dryness, and reconstituted in 25 μL in 97:3 acetonitrile: water +0.1 percent formic acid.

### 4.4. Liquid Chromatography

All the chromatographic steps were used with ULC/MS solvents. Each sample was loaded and analyzed using split-less nano-Ultra Performance Liquid Chromatography 10 kpsi nanoAcquity; Waters, Milford, MA, USA). The mobile phase was a) H_2_O + 0.1% formic acid and + 0.1% formic acid B). Sample desalination was carried out online with a reversed-phase symmetry C18 trapping column (180 μm internal diameter, 20 mm length, 5 μm particle size; water). Peptides were then separated by a nano-column of T3 HSS (internal diameter 75 μm, length 250 mm, partition size 1.8 μm; water) with 0.35 μL/min. The peptides were eluted from the column to the mass spectrometer by the following gradients: 4% to 30% B in 155 min, 30% to 90% B in 5 min, maintained at 90% for 5 min and re-started.

### 4.5. Mass Spectrometry

NanoUPLC-MS/MS was used by connecting online to a quadrupole orbitrap mass spectrometer (Q Exactive Plus, Thermo Scientific) using a FlexIon nanospray device via a nanoESI emitter (10 μm tip; New target; Woburn, MA, USA) (Proxeon, San Jose, CA USA). Data has been acquired by a Top10 method in DDA mode. MS1 resolution has been set to 70,000 (400 *m*/*z*) and the maximum injection time has been set at 60 s. The MS2 resolution and maximum injection time of 120 ms were set to 17,500.

### 4.6. Data Analysis

MaxQuant v1.6.0.16 was used and processed raw data. The data searched with Andromeda search engine in UniprotKB and added with common laboratory protein contaminants. The specificity of the enzyme was set for trypsin and two missed cleavages were permitted. Cysteine carbamidomethylation was kept fixed and variable changes were made in methionine oxidation, N or Q deamidation, and S, T or Y phosphorylation. A maximum mass difference of 4.5 ppm and fragmented ions, a maximum mass difference of 20 ppm, was searched for peptide precursor ions. The decoy database strategy was used to filter peptide, protein and site identifications at an FDR of 1 percent. The minimum length of peptides was 7 amino acids with a minimum Andromeda score of 40. Peptide identifications have been spread over samples using the intermediate match option checked. Searches have been conducted using the selected label-free quantification option. Perseus v1.6.0.7 was used to calculate the quantitative comparisons. Decoy hits and phosphosites with no probability for at least 75 percent of the site, or two valid values in at least one experimental group, were filtered. After a logarithmic transformation, a *t*-test student was used to identify significant differences between biological replicas. Fold changes based on the geometric means of the case vs. control samples ratio were calculated.

### 4.7. Gene Ontology (GO) Analysis

The significantly altered phosphopeptides with up- and downregulated sites on CLA supplementation were classified based on GO Biological Process and Molecular Structure to examine functional enrichment through phosphoproteins using the BINGO functional annotation method in Cytoscape. For each of the control and CLA-supplemented groups, the GO terms were filtered to significantly enriched groups with a *p* value of <0.005 and a minimum of three representative genes in each class. The filtered *p* values were visualized as bar graphs based on GO terms, molecular function, and KEGG pathways. The pathway annotation was obtained from the Reactome pathway database for metabolic enzymes and the Foam tree for lipid metabolism.

### 4.8. Analysis of Hierarchical Clustering

Using the IDEP.91 server tool with an average linkage clustering and Kendall’s tau distance measurement process, unsupervised hierarchical clustering of individual samples and the detected differentially expressed proteins was carried out based on their relative peptide abundances in each sample. The heatmap and PCA were developed and visualized the peptide expression with dendrograms displaying clustering results. Means for the biological samples have been measured and fold-changes have been determined by dividing the mean strength of the CLA samples handled by the control samples for each peptide. Using the log2 function, the fold change was transformed so that the data is centered on zero, while for volcano plot scaling, the Benjamini–Hochberg-corrected *p* value was −log10 transformed.

### 4.9. Kinase-Substrate Prediction

The iGPS 1.0 and NetworKIN 3.0 algorithms was used to predict the possible kinase-substrate relationships and thus kinase activity in CLA and control groups. The significant upregulated and downregulated peptides were kept in PhosPEP format and serene-threonin kinases were found at a false positive rate (FPR) of 10%. Statistical enrichment of all detected phosphopeptides was carried out using a hypergeometric test and kinase-substrate relationships used as an iGPS background. The NetworKIN works by deriving from the NetPhorest Atlas for kinases and close ranges of protein–protein interactions were combined in order to achieve NetworKIN values that are represented as probability ratios. The phosphopeptides were matched with the predictions for the phosphosites expressed differently. In order to gain confidence in the predicted results, only those with NetworKIN score > 2 or NetPhorest probability > 0.1 were filtered before statistical enrichments were obtained by means of hypergeometric distribution. Only kinases enriched by a *p*-value reduction below 4–10 were considered to be significantly altered. Additional kinase-substrate enrichment analysis based on the average abundances (log2 fold changes) of phosphorylated substrates was performed for those kinases that were expected to be significantly enriched in both upregulated and downregulated substrates to assign the activity of kinase to both the control and CLA-supplemented AT.

### 4.10. Network Analysis of Phosphopeptides

The interactions of phosphoproteins were explored using the STRING database and GeneGO MetaCore. The pairwise interactions of differentially mediated up- and downregulated phosphoproteins were downloaded by loading into the STRING database. With a combined score > 0.4, STRING records the medium confidence interaction scores of the pairs. The threshold was increased to 0.6 in this study to maintain high trust interactions. Additionally, we derived path-based functional interaction from Reactome and direct interactions from the MetaCore software suite Based on experimental data, the interactions recorded in both of these instruments are manually curated, and thus all the interactions collected have been retained. In order to generate integrated network, all the interactions collected were loaded as SIF (simple interaction file) into Cytoscape software and merged with each other. By removing all duplicate entries, redundant proteins were eliminated. The properties of network were explored by Network Analyzer and densely linked clusters by Cluster ONE in Cytoscape. The functional enrichment was performed using Reactome pathways in each of the clusters. The pathways related to each protein were collected from Kyoto Encyclopedia of Genes and Genomes (KEGG) for metabolic network mapping, and the schema was built using ‘AMPK signaling’, lipolysis in adipocytes, ‘Insulin signaling, cAMP signaling, mTOR signaling’, and ‘Gluconeogenesis’ components of the pathway map. Upstream kinases were obtained from literature for establishing regulations and functional consequences. Finally, in a graphical way, all the information was visualized.

### 4.11. Quantitative Real-Time PCR of AT Samples

The gene expression was determined in omental and subcutaneous AT samples. RNA was extracted by collecting 40 mg of AT samples and homogenized in 1 mL of lysis solution using RNA isolation kit (Qiagen, Hilden, Germany). RNA quality was assessed at 260/280 ratio and above 1.85. cDNA was synthesized using cDNA reverse transcription kit (Applied Biosystems, Foster City, CA, USA) and quantitative detection of mRNA was done by using Real-time PCR (StepOnePlus; Applied Biosystems) and SYBR green PCR mix (Invitrogen, Carlsbad, CA, USA). The list of primers is presented in Supplementary Table S3. The relative quantity (RQ) values were normalized to one AT sample and divided by the average RQ of the gene and used in statistical analysis.

### 4.12. Western Blot Analysis

To examine proteins related to AT metabolism, 20 µg of each sample with Laemmli loading buffer was resolved by SDS-PAGE under reducing conditions, and transferred onto a nitrocellulose membrane with antibodies: MGLL (1:1000, ab24701, Abcam Biotech, Cambridge, UK), β-actin (1:1000, #ab46805, Abcam Biotech), CB1 (1:200, #ab23703, Abcam Biotech), CB2 (1 µg/mL, #ADI-905-820-100, Enzo Life Sciences, Farmingdale, NY, USA), FASN (1:2000, #99359, Abcam Biotech), DAGLA (0.3 µg/mL, #oaeb01139, Aviva Systems Biology, San Diego, CA, USA) and FAAH (1 μg/mL, #ARP33121_P050, Aviva Systems Biology) in AT samples. An enhanced protein chemiluminescence reaction was used for dilution by 1:10,000 Goat Antirrabbit Horseradish Peroxidase secondary antibody (111-035-003, Jackson Immunoresearch Laboratories, Inc., West Grove, PA, USA). Data were processed and analyzed using ImageJ software for densitometry (NIH, Bethesda, MD, USA). Chemiluminescent signals were measured after at least 5 consecutive exposure times to determine the linear range of signal intensity of each anticorder, to ensure quantitative data were obtained. Specific band signals to β-actin were normalized.

### 4.13. Statistical Analyses

Statistical analyses were made using SAS 9.4 for Windows (SAS Institute Inc. Cary, NC, USA). Performance data and plasma concentrations of metabolites were analyzed by MIXED procedure using repeated-measures ANOVA containing CLA (level: yes, no), time (levels: day or week relative to calving), block (levels: 1 to 5), and the interaction (CLA vs. time) as fixed effects. The calving interval and projected milk yield during the second lactation were used as covariates. Repeated measures on each cow were considered by using the repeated statement of the MIXED procedure with compound symmetry (timeline d or wk) covariance structure. The least squares means (LSM) and their standard errors (SE) were computed for each fixed effect in the ANOVA model to display the results. Additionally, group differences in these LSM were tested using the Tukey-Kramer procedure. All differences with *p* < 0.05 were considered significant.

Phosphoproteomics data were analyzed by 2-way ANOVA using Stat model of Python 3.6.4 to study the effects of treatment (CON vs. CLA), fat depot (OM vs. SC) and the interaction between them. The abundance of the differential proteins was determined by *p* < 0.05 and FC ± 1.5. The data were analyzed using the ratios of the observed means calculated and the outlines measured for each significant protein identified [64]. The immunoblot-determined protein abundance and mRNA expression were analyzed by the GLM procedure version 9.2 of SAS.

## 5. Conclusions

This comprehensive study of subcutaneous and omental AT phosphoproteome in dairy cows following CLA supplementation reveals previously unknown phosphorylation sites, suggesting increased lipid turnover in AT. The phosphoproteome results obtained in CLA-supplemented AT shed light on the altered protein signatures activated by lipolysis and lipogenesis. In this study, we highlighted the mechanisms of metabolic alterations in response to the stimuli and identified that activation of early signaling cascades including insulin signaling, MAPK, lipolysis in adipocytes, and AMPK signaling; it seems that more lipogenesis occurs than lipolysis in CLA-supplemented dairy cows in AT. We suggest that phosphoproteomic analysis of AT provides a molecular view in lipid metabolism that underlies the metabolism of AT depots and exposes the regulation of proteins in AT, retaining energy homeostasis.

## Figures and Tables

**Table 1:** Animal performance data, milk production and components, plasma metabolites, plasma hormones and plasma conjugated linoleic acid (CLA) content at wk 8 postpartum of cows abomasally supplemented either with: coconut oil [control, (CON); *n* = 5] or conjugated linoleic acid (CLA; *n* = 5) from d 63 antepartum until slaughter on d 63 postpartum.

	Treatment ^1^		*p*-Value
Variable	CON	CLA	
Performance			
DMI, kg DM/d	20.7 ± 1.6	17.6 ± 1.3	0.19
Energy intake, MJ NE_L_/d	148 ± 11	125 ± 10	0.17
Energy balance, MJ NE_L_/d	−26.0 ± 9.2	6.5 ± 7.9	0.04
Body weight, kg	654 ± 31	611 ± 26	0.32
Body condition score	2.69 ± 0.19	2.94 ± 0.18	0.39
Back fat thickness, mm	8.66 ± 1.54	11.80 ± 1.42	0.21
Milk production and components			
Milk yield, kg/d	42.9 ± 2.9	37.9 ± 2.5	0.23
ECM, kg/d	43.2 ± 3.2	26.2 ± 2.8	0.01
Milk fat content, %	4.53 ± 0.27	1.73 ± 0.23	<0.001
Milk fat yield, kg	1.81 ± 0.17	0.64 ± 0.14	<0.01
Milk protein content, %	3.07 ± 0.10	2.90 ± 0.08	0.24
Milk protein yield, kg	1.31 ± 0.07	1.10 ± 0.06	0.06
Milk lactose content, %	4.75 ± 0.07	4.81 ± 0.06	0.52
Milk lactose yield, kg	2.03 ± 0.16	1.83 ± 0.13	0.34
Milk urea, mg/L	140 ± 9	124 ± 8	0.21
SCC × 1000/mL	2 ± 220	287 ± 188	0.35
Metabolites			
NEFA, µmol/L	384 ± 44	145 ± 37	0.01
Triglycerides, mmol/L	0.10 ± 0.01	0.08 ± 0.01	0.26
Cholesterol, mmol/L	5.46 ± 0.66	4.92 ± 0.56	0.55
Glucose, mmol/L	3.34 ± 0.23	3.32 ± 0.19	0.96
BHB, mmol/L	0.59 ± 0.13	0.69 ± 0.11	0.57
Hormones			
Insulin, µg/L	0.23 ± 0.09	0.48 ± 0.07	0.07
Glucagon, ng/L	144 ± 12	109 ± 10	0.06
GH, µg/L	7.31 ± 1.82	3.79 ± 1.56	0.19
IGF-1, µg/L	54.6 ± 24.7	123.0 ± 21.1	0.08
IGFBP-2, µg/mL	6.62 ± 1.20	3.76 ± 1.10	0.15
IGFBP-3, µg/mL	2.33 ± 0.38	3.69 ± 0.35	0.06
IGFBP-4, µg/mL	1.35 ± 0.36	1.36 ± 0.33	0.99
Cortisol, nmol/L	21.7 ± 6.4	22.0 ± 5.4	0.97
Leptin, ng/mL	3.70 ± 2.71	9.64 ± 2.32	0.14
Adiponectin, µg/mL	22.3 ± 2.5	18.5 ± 2.1	0.28
Plasma CLA content			
C18:2 *cis*-9, *trans*-11 CLA, g/100 g fatty acids	0.06 ± 0.03	0.56 ± 0.03	<0.001
C18:2 *trans*-10, *cis*-12 CLA, g/100 g fatty acids	0.00 ± 0.02	0.19 ± 0.01	<0.001

^1^ Values represented as least squares means with SE. DMI = Dry matter intake; ECM = energy corrected milk; SCC = somatic cell count; NEFA = nonesterified fatty acids; BHB = β-hydroxybutyrate; GH = growth hormone; IGF-1 = insulin-like growth factor-1; IGFBP-2, 3, 4 = insulin-like growth factor binding protein-2, 3, 4, respectively; statistical differences were analyzed using the MIXED procedure using repeated measures of SAS, *p* ≤ 0.05 was considered significant.

**Table 2:** Body weight (BW), hot carcass weight (HCW), cold carcass weight (CCW), adipose depot weights, and their proportion of BW at slaughtering at wk 9 postpartum for cows daily abomasally supplemented either with coconut oil (CON; *n* = 5) or conjugated linoleic acid (CLA; *n* = 5) from d 63 antepartum until slaughter on d 63 postpartum.

	Treatment ^1^		*p*-Value
Variable	CON	CLA	
BW, kg	649 ± 30	614 ± 25	0.40
HCW			
Weight, kg	261 ± 19	266 ± 16	0.86
Proportion of BW, %	40.1 ± 1.3	43.3 ± 1.2	0.12
CCW			
Weight, kg	254 ± 19	259 ± 16	0.85
Proportion of BW, %	39.0 ± 1.4	42.2 ± 1.2	0.14
Subcutaneous adipose			
Weight, kg	0.72 ± 0.11	0.67 ± 0.10	0.73
Proportion of BW, %	0.11 ± 0.01	0.11 ± 0.01	0.96
Retroperitoneal adipose			
Weight, kg	5.96 ± 1.41	7.30 ± 1.21	0.49
Proportion of BW, %	0.90 ± 0.21	1.17 ± 0.18	0.35
Omental adipose			
Weight, kg	4.46 ± 1.01	6.97 ± 0.87	0.11
Proportion of BW, %	0.66 ± 0.14	1.12 ± 0.12	0.04
Mesenteric adipose			
Weight, kg	4.48 ± 0.86	4.95 ± 0.74	0.68
Proportion of BW, %	0.68 ± 0.11	0.78 ± 0.10	0.52
Total adipose ^2^			
Weight, kg	15.6 ± 3.0	19.9 ± 2.5	0.31
Proportion of BW, %	2.35 ± 0.38	3.18 ± 0.32	0.14

^1^ Values are presented as least squares means with SE. ^2^ Sum of subcutaneous, retroperitoneal, omental, and mesenteric adipose. BW = body weight; HCW = hot carcass weight; CCW = cold carcass weight. Statistical differences were analyzed by the MIXED procedure using repeated measures of SAS, *p* ≤ 0.05 was considered significant.

**Table 3:** Average relative gene expression in CON and CLA AT from cows.

RQ	CON	CLA	*p*-Value CLA ^1^ vs. CON ^2^	*p*-Value SC ^3^ vs. OM ^4^	*p*-Value Interaction trt *AT Depot
*LIPE*	0.82 ± 0.16	1.37 ± 0.16	0.03	0.95	0.47
*FASN*	0.08 ± 0.27	1.12 ± 0.27	0.01	0.14	0.16
*PLIN*	0.74 ± 0.17	1.04 ± 0.17	0.22	0.06	0.72
*PTGS2*	0.9 ± 0.09	0.82 ± 0.09	0.56	0.04	0.31
*DAGLA*	1.05 ± 0.21	1.68 ± 0.21	0.05	0.05	0.91
*MGLL*	1.4 ± 0.10	1.28 ± 0.10	0.46	<0.0001	0.24
*FAAH*	0.67 ± 0.08	0.71 ± 0.08	0.74	0.002	0.93
*CNR1*	1.85 ± 0.23	1.13 ± 0.23	0.04	0.02	0.16
*NAPEPLD*	0.53 ± 0.07	0.67 ± 0.07	0.15	0.63	0.51
*CNR2*	2.86 ± 0.17	2.4 ± 0.17	0.08	<0.0001	0.09

^1^ CLA—Conjugated linoleic acid; ^2^ CON, control; ^3^ SC, subcutaneous AT; ^4^ OM, omental AT. Statistical differences were analyzed by the GLM procedure of SAS, * *p* ≤ 0.05 was considered as significant.

## Data Availability

The data presented in this study are available in supplementary material.

## Supplementary Materials

The following are available online at https://www.mdpi.com/1422-0067/22/6/3227/s1, Figure S1: AMPK signaling with up (pink color) and downstream (red color) kinases in CLA supplemented AT was generated by Ingenuity Pathway Analysis (IPA) software. Different enzymes were represented in circles, inverted triangles and squares in the pathway. The enzymes not involved in the pathway were colorless, Figure S2: Insulin signaling with up (pink color) and downstream (red color) kinases in CLA supplemented AT was generated by Ingenuity Pathway Analysis (IPA) software. Different enzymes were represented in circles, inverted triangles and squares in the pathway. The enzymes not involved in the pathway were colorless, Figure S3: Foam tree analysis of biological pathways by phospho proteins in Adipose tissue of dairy cows. From the mechanisms, metabolism of lipids was selected and the pathways involved were identified. Among these, lipolysis, lipogenesis, insulin signaling, AMPK signaling and mTOR signaling pathways have significant role in the lipid metabolism, Figure S4A: Immunoblots of CB2, FAAH, MGLL and DAGLA proteins in subcutaneous AT of CON vs. CLA dairy cows (*n* = 1–4 replicates for CON; *n* = 5–9 replicates for CLA). Expression levels of CB2, FAAH, MGLL, DAGLA proteins in subcutaneous AT of CON vs. CLA. Actin was used as control to quantify the proteins, Figure S4B: Immunoblots of CB2, FAAH, MGLL and DAGLA proteins in omental AT of CON vs. CLA dairy cows (*n* = 1–4 replicates for CON; *n*= 5–9 replicates for CLA). Expression levels of CB2, FAAH, MGLL, DAGLA proteins in omental AT of CON vs. CLA. Actin was used as control to quantify the proteins, Table S1: Relative gene expression in OM and SC AT of CON and CLA cows, Table S2: Location, type, name, Up and down regulation of protein kinases in insulin and mTOR signaling pathways of CON vs. CLA dairy cows, Table S3: List of primers used for mRNA expression in CON vs. CLA AT, Table S4: Immunoblot abundance of lipid-metabolism proteins in SC and OM AT of CLA vs. CON cows, Table S5: Amounts of daily abomasally infused supplements to dairy cows supplemented either with coconut oil (CON; *n* = 5) or conjugated linoleic acid (CLA; *n* = 5) from d 63 antepartum until slaughter on d 63 postpartum, Table S6: Ingredients and chemical compositions of the diets provided to cows abomasally supplemented either with coconut oil (CON; *n* = 5) or conjugated linoleic acid (CLA; *n* = 5) from d 63 antepartum until slaughter on d 63 postpartum.
